# Case in Surgery

**Published:** 1854-11

**Authors:** J. C. Hughes

**Affiliations:** Professor of Surgery in the Medical Department of the Iowa University


### CASE IN SURGERY.

BY J. C. HUGHES, M. D.,

Professor of Surgery in the Medical Department of the Iowa University.

Case I.—*Operation for Complicated Hair Lip.*—Thos. McGuire, aged 18 years, presented himself, Nov. 29th, at the Hospital for operation. I found upon examination, his condition to be as follows: The external fissure, reaching continuously from the lower edge of the lip on the right side of the corresponding nostril, which was widely separated, being about an inch and a quarter wide at its lower or labial portion, and terminating in the nostril by an opening three quarters of an inch wide. There was also an arrest of development in the superior maxilliary, the fissure in the bones being partially filled by irregular teeth projecting horizontally from the left side. The complication with cleft palate which existed, extended through both the hard and soft structures, exceeding in width half an inch.

Preparatory to the operation for this repulsive deformity, I found it necessary to remove a number of the teeth which would obstruct the anticipated union of the soft parts. This having been done, forcible pressure was applied for some days to the projecting structures, for the purpose of obtaining a smooth basis for the parts about to be supported by them. These points having been accomplished, the patient was brought before the class for operation.—After administering the chloroform, (given at his own request,) I took hold of one side of the lip, separating it freely with the scalpel from its attachment with the gum, then adopting the same course with the other, both were made free; which is necessary to success, by diminishing the subsequent strain on the line of union. Then, to make a fresh surface upon the edges, I introduced a tenotomy knife through the lip at the nasal angle upon the left side of the fissure, making an incision obliquely outwards nearly as far as the labial border, when the direction was changed toward the fissured part. The same course was adopted with the opposite side, so that when the lips were brought together the projection formed by the angular incision would supply the deficiency in the lip, which would otherwise ensue. The angles of the lips were now brought upon the same level and accurately adjusted by passing a ligature through their lower edges. I then introduced a needle, bringing the raw edges in direct contact by the application of the twisted suture. The ligature was now removed and a second needle introduced, which was also retained by the twisted suture; after this the muscles were controlled by the isinglass plaster, which completed the operation.

The parts were kept at perfect rest for forty-eight hours; adhesion by the first intention took place, and on the third day the first needle was removed, without removing the suture; on the fourth day the second. The sutures and isinglass plasters were allowed to remain some eight days. I had thought of carrying out in this case the mode of operating in Labial Tissue which originated and for some years has been adopted by Prof. Ackley, of Cleveland, Ohio. The lips are brought together either by hair-lip needles, or by a needle armed with a strong thread. This done, a fine needle armed with a very fine silk thread is passed very superficially through the contiguous margins, so as only to include the epidermis, and by numerous stitches, 20, 30, or even 40, as the case may be, are brought very accurately and smoothly together, which has the effect to prevent, or at least, lessen, the liability to prolabial chasm. But, as you will perceive, perfect and favorable union was the result in the mode adopted.

The fissure of the cleft palate is so very wide, any attempt to remedy it by a surgical operation is unfortunately out of the question. A metalic plate which is being fitted to the part by a dentist of this city, will obviate the difficulty as effectually as art can do it.

The favorable effect of the operation upon the appearance of the patient has been remarkable. The deformity had existed during his life time, and was accompanied by so marked a distortion of his features as to present an appearance almost revolting. At this time the nostril, formerly widely separated, is of a natural shape and width; the upper lip is of a uniform width and appearance, and the countenance of the patient shows no remains of his former misfortune. This contrast is more fully shown by the wood cuts, which give his appearance both before and after the operation. (These cuts were taken from daguerreotypes now in my possession.) His enunciation, also formerly so indistinct and imperfect that strangers could not understand his half-formed language, is much more clear and distinct, and can be readily understood.

